# Delirium in Australian Hospitals: A Prospective Study

**DOI:** 10.1155/2013/284780

**Published:** 2013-09-12

**Authors:** C. Travers, G. J. Byrne, N. A. Pachana, K. Klein, L. Gray

**Affiliations:** ^1^Queensland Dementia Training Study Centre, Queensland University of Technology, Kelvin Grove, QLD 4059, Australia; ^2^Centre for Psychiatry & Clinical Neuroscience, The University of Queensland, Royal Brisbane and Women's Hospital, Herston, QLD 4029, Australia; ^3^School of Psychology, The University of Queensland, Brisbane, QLD 4072, Australia; ^4^Queensland Clinical Trials & Biostatistics Centre, School of Population Health, University of Queensland, The Princess Alexandra Hospital, Woolloongabba, QLD 4102, Australia; ^5^Centre for Research in Geriatric Medicine, The University of Queensland, The Princess Alexandra Hospital, Woolloongabba, QLD 4102, Australia

## Abstract

*Objectives*. Australian data regarding delirium in older hospitalized patients are limited. Hence, this study aimed to determine the prevalence and incidence of delirium among older patients admitted to Australian hospitals and assess associated outcomes. *Method*. A prospective observational study (*n* = 493) of patients aged ≥70 years admitted to four Australian hospitals was undertaken. Trained research nurses completed comprehensive geriatric assessments using standardized instruments including the Confusion Assessment Method to assess for delirium. Nurses also visited the wards daily to assess for incident delirium and other adverse outcomes. Diagnoses of dementia and delirium were established through case reviews by independent physicians. *Results*. Overall, 9.7% of patients had delirium at admission and a further 7.6% developed delirium during the hospital stay. Dementia was the most important predictor of delirium at (OR = 3.18, 95% CI: 1.65–6.14) and during the admission (OR = 4.82; 95% CI: 2.19–10.62). Delirium at and during the admission predicted increased in-hospital mortality (OR = 5.19, 95% CI: 1.27–21.24; OR = 31.07, 95% CI: 9.30–103.78). *Conclusion.* These Australian data confirm that delirium is a common and serious condition among older hospital patients. Hospital clinicians should maintain a high index of suspicion for delirium in older patients.

## 1. Introduction

Delirium is common among older people admitted to acute hospitals, and this problem is likely to increase in the near future as a consequence of population ageing and the greater likelihood of being admitted to hospital with older age [1–3]. Reported prevalence rates for delirium range from 10% to 31% at admission and between 3% and 29% during admission in medical inpatients while considerably higher rates have been reported for older patients following cardiac and hip fracture surgery where rates of 47%–53% have been reported [4, 5] and in older patients admitted to the intensive care unit where rates in excess of 80% have been reported [6]. The consequences of delirium can be serious, and delirium is associated with longer admissions, increased morbidity and mortality, a greater likelihood of admission to institutional care, and increased hospital costs [1, 7].

To ensure the adequate provision of hospital services for these patients, accurate data, regarding the prevalence and incidence of delirium, and risk factors are required. The majority of studies conducted to date have been in Europe and the USA, and few Australian studies have examined delirium in older hospitalised patients [1, 8–11]. The lack of such research was identified by the authors of the first set of national clinical practice guidelines for the management of delirium who indicated a need for further epidemiological and risk factor research within Australia [12]. Because there are important differences in Australia's health and aged care systems in comparison to other countries, the applicability of risk prediction models developed overseas to the Australian context is unclear. For example, Australia's universal healthcare system (Medicare) allows for healthcare that is more accessible than in some other countries, while Australia ranks more highly on a number of healthcare system quality indicators, including chronic care, than several other developed nations [13]. These factors may influence hospitalization patterns for older Australians including the prevalence and incidence of delirium in general hospitals. In particular, older Australians presenting to hospital might be at reduced risk for delirium in comparison with older persons from other countries with less comprehensive access to healthcare.

Thus, a key aim of the present study was to accurately determine the prevalence and incidence of delirium in older patients admitted to general medical, general surgical, and orthopedic wards (infrequently undertaken in a single study) in general hospitals in Queensland. These data will supplement existing Australian data and provide essential information for the planning and provision of hospital services for older patients. Additional study aims were to describe the demographic and clinical characteristics of patients with delirium admitted to each ward type and assess the contribution of delirium, relative to other important patient characteristics, to adverse outcomes. 

## 2. Method

### 2.1. Study Design

This was a substudy of a prospective observational study of a sample of patients aged ≥70 years admitted to four general hospitals in south-east Queensland, Australia, in 2008–2010. Comprehensive details regarding participants and methods have been published elsewhere [14] and are briefly reiterated here.

### 2.2. Subjects

Patients aged ≥70 years admitted to selected wards in four hospitals and expected to remain in hospital for at least 48 hours (to allow an adequate observation period and to complete assessments) were potentially eligible to participate in the study. The hospitals included two major university teaching hospitals (>700 beds) in Brisbane, Queensland, and two smaller district hospitals (250–280 beds) near Brisbane. The wards included general medical and general surgical wards from all four hospitals and orthopedic wards from the two larger hospitals. Patients were excluded if they were transferred to a study ward from another hospital or ward, had been admitted more than 48 hours previously, or were immune compromised and hence in isolation, or where death was imminent (to respect the patient's privacy). 

### 2.3. Ethical Approval

Ethical approval was obtained from the ethics committee of the University of Queensland and from each hospital's Human Research Ethics Committee, prior to commencement. Informed written consent was obtained from each patient (or their legal guardian) prior to participation in the study. 

### 2.4. Procedure & Assessments

Experienced research nurses performed comprehensive patient assessments and administered standardized assessment instruments to patients within 48 hours of admission. If a patient had surgery requiring a general anesthetic (GA) within 36 hours of admission, the assessment was completed 72 hours following surgery. The interRAI Acute Care (interRAI AC) instrument was used to obtain comprehensive information (it encompasses 12 domains including cognition, communication, mood and behavior, activities of daily living (ADL), and medical diagnoses) about each patient's physical and cognitive status and psychosocial functioning prior to the onset of the illness/condition, at admission (based on the patient's first 24 hours in the ward) and at discharge [15, 16]. The Mini-Mental Status Examination (MMSE) [17] was administered to assess patient's cognitive functioning, and patients were screened for delirium using the Confusion Assessment Method (CAM) [18]. Additional assessments included the Vulnerable Elders Survey-13 (VES-13) [19], a 13-item self-report questionnaire that identifies vulnerable older people, defined as “older people at increased risk of death or functional decline.” The patient's relative or caregiver was also asked to complete the 16-item Informant Questionnaire for Cognitive Decline in the Elderly (IQCODE) [20], and the Neuropsychiatric Inventory (NPI-Q) [21], to measure the frequency and severity of psychological and behavioral problems that the patient may have displayed.

All patients were reviewed daily throughout their hospital stay by the research nurse to determine whether any adverse events, including falls and acute change in mental status (defined as acute change in attention, orientation, or cognition), had occurred in the previous 24 hours (data regarding adverse events that occurred on weekends were collected the following Monday). If an acute change in the patient's mental status was documented during the hospital stay, the CAM was readministered. The CAM and interRAI AC were the only instruments administered on more than one occasion.

The Charlson Comorbidity Index (CCI) [22] was used to assess the severity of chronic comorbidity (age adjusted) for each patient using a Microsoft Excel Macro specifically designed for this purpose [23]. The CCI was calculated using all diagnoses (the primary diagnosis for the admission and all other active diagnoses) that were recorded on the interRAI AC. The research nurse also telephoned each patient (or caregiver) 28 days following his/her discharge from hospital, to determine whether any adverse events including death, readmission to a general hospital, or admission to a nursing home had occurred in the immediate postdischarge period. Finally, each patient's principal diagnosis (Australian Refined Diagnosis Related Groups Version 5.1 (AR-DRG v5.1)) and associated diagnoses for the admission were obtained from the Queensland Health Department's administrative database following their discharge.

### 2.5. Diagnosis of Cognitive Impairment

#### 2.5.1. Dementia

Following the patient's discharge, chart reviews were performed by two independent expert physicians (specialist geriatricians and psychogeriatricians) to determine, via consensus, whether the patient was likely to have dementia prior to the onset of their current illness/condition. The majority of cases were reviewed and complete details regarding case selection have been described elsewhere [14]. Physicians were asked to determine whether patients met the criteria common to the various types of dementia listed in DSM-IV (criteria A and B, and the criterion indicating that “the deficits do not occur exclusively during the course of a delirium”) [24], in which case dementia was considered likely. If the patient did not meet these criteria, dementia was considered unlikely. To make this decision, physicians had access to all available clinical information, including the patient's chart with the referral letter from the patient's general practitioner (primary care physician), and discharge summaries completed by hospital medical staff, as well as all assessments completed by the research nurse with the exception of the interRAI AC Cognition items (Section D) (reserved for validity studies). Physicians were not asked to specify the dementia type [24].

#### 2.5.2. Delirium

Physicians were also asked to determine whether patients met the DSM-IV criteria for delirium, at admission or subsequently. If the patient was considered to meet all the three DSM-IV core criteria (A, B, and C), delirium was considered likely; otherwise delirium was considered unlikely. Physicians were not asked to specify the etiology [24]; however, if a patient was considered to have had delirium, they were asked to indicate whether it had resolved by discharge. 

### 2.6. Data Analysis

Differences between groups were assessed using the chi-squared statistic for categorical data and Student's *t*-test for continuous variables. To control for potential inflation of type I error rate secondary to multiple comparisons, the *P* values (reported in Tables 1 and 2) were adjusted according to the method recommended by Benjamini and Yekutieli [25]. Multivariate logistic regression analyses were performed, using the Forward Likelihood Ratio entry method, to identify independent variables associated with delirium at admission, delirium during the admission (incident delirium), and persistent delirium. The contribution of delirium, relative to other important patient characteristics, to adverse outcomes including falls in hospital, in-hospital mortality, discharge to a higher care level (admission to a nursing home when the patient had previously lived in the community or transfer from low to high nursing home care following hospitalization), and death within 28 days following discharge was also assessed. Odds ratios (OR) and 95% confidence intervals (CI) were calculated. Variables assessed as possible predictors of delirium at admission included hospital site, age, a dementia diagnosis, gender, CCI scores, interRAI AC ADL scale scores (premorbid ability; dichotomized into Independent versus not independent), VES-13 scores, and vision and hearing impairment. In addition to these variables, length of stay, delirium at admission, whether the patient had undergone a GA, and the ward type admitted to were also assessed as possible predictors of incident delirium and persistent delirium while delirium superimposed on dementia was also examined as a potential predictor of adverse outcomes. Negative binomial regression was performed to investigate the association between these variables and length of stay. Statistical analyses were performed using SPSS for Windows version 18.0 [26]. 

## 3. Results

Of 768 patients aged ≥70 years admitted to the study hospitals, 733 met the eligibility criteria and 499 patients (68.1%) consented to participate. Six patients (1.2%) withdrew following initial consent, leaving data for 493 patients available for analysis. Reasons for nonarticipation have been described elsewhere [14]. There were no significant differences between study participants and those who either declined to participate in the study or withdrew, in terms of age (*t*(1) = 1.16, *P* = 0.25) or length of admission (*t*(1) = 0.21, *P* = 0.94). 

Participant's mean age was 80.4 years (SD = 6.5 years, range 70–99 years) and 58.4% were females (*n* = 288). The majority were admitted to general medical wards (*n* = 294; 59.6%), while 140 (28.4%) were admitted to general surgical and 59 (12.0%) to orthopedic wards. The average MMSE score was 24.8 (SD = 4.9; range 0–30). One hundred and two (20.7%) patients underwent a procedure requiring a GA, and of those 59 (57.8%) were assessed by the research nurse prior to surgery (i.e., within the first 48 hours of admission) while 42.2% (*n* = 43) were assessed following the procedure. 

Independent physicians reviewed 75.5% of cases (*n* = 372). Following review, 102 (20.7%) were considered likely to have dementia (of patients with an MMSE score between 27 and 30, 4 (1.9%) were considered to have dementia). The overall prevalence of delirium at admission was 9.7% (*n* = 48) while an additional 34 (7.6%) patients without delirium at admission developed incident delirium during their hospital stay (of patients with an MMSE score between 27 and 0, 1 (0.5%) had delirium at admission and 5 (2.4%) developed incident delirium). A further 48 patients (9.7%) displayed one or more symptoms of delirium (as measured by the CAM) suggestive of subsyndromal delirium but did not exhibit all three DSM-IV core criteria for delirium. Thirty-nine (7.9%) patients were considered to have delirium superimposed on dementia, representing 47.6% of those with delirium. Of patients with delirium, the delirium was considered to persist at discharge in 36 cases (43.9%). Only 2/82 (2.4%) of those with delirium had an AR-DRG code for delirium recorded in official hospital records. There was no significant difference in the prevalence or incidence of delirium according to ward type.

Key demographic features, clinical characteristics, and assessment results of patients with and without delirium at admission and incident delirium are shown in Table 1. Patients with delirium at admission were significantly more likely to have dementia (23.5%) than patients without dementia (6.1%), while the rate of incident delirium in the two groups was 19.2% and 5.2%, respectively. Patients with delirium were also significantly older, were more frequently admitted to hospital from a nursing home, and performed more poorly on most assessment measures administered compared to patients without delirium. Fewer differences were evident between patients with incident delirium and those who did not develop delirium during the admission. Compared to patients without delirium, patients with delirium, either at or during the admission, had significantly longer hospital stays and more falls in hospital. Higher in-hospital mortality rates were evident for patients with incident delirium, while higher mortality rates at 28 days after discharge were evident in patients who had delirium at any time (either at or during the admission) compared to patients without delirium (see Table 2). There were no significant differences in any adverse outcome associated with delirium or in the persistence of delirium at discharge across the three ward types; hence data are presented as aggregated data across the ward types.

Results of the multivariate logistic regressions are shown in Table 3. A diagnosis of dementia together with older age independently predicted delirium at admission, while dementia and longer hospital stays independently predicted incident delirium. Delirium at and during the admission significantly predicted in-hospital mortality while no variable emerged as a significant predictor of persistent delirium. Results of the negative binomial regression showed five variables: incident delirium (*P* < 0.001), having a procedure with a GA (*P* < 0.001), delirium at admission (*P* = 0.001), higher VES-13 scores (*P* = 0.029), and CCI scores (*P* = 0.018), which were significantly associated with longer hospital stays.

## 4. Discussion

This study provides new data regarding the prevalence and incidence of delirium in older patients admitted to Queensland hospitals and adds to the small, but growing, body of Australian data regarding delirium in this setting. In this study, 9.7% of older patients admitted to general hospitals had delirium at admission while a further 7.6% developed incident delirium, with considerably higher rates in patients with dementia (23.5% and 19.2%, resp.). Patients with delirium experienced more serious adverse outcomes than patients without delirium including longer hospital stays, more falls in hospital, and higher mortality rates, both in-hospital and at 28 days after discharge, and there was no difference in the rate of adverse outcomes across the three ward types. After controlling for potentially confounding variables, delirium at and during the admission was found to independently predict in-hospital mortality while delirium at admission was found to be marginally significant as an independent predictor of falls in hospital. In this study, the relationship between incident delirium and in-hospital mortality was exceptionally high (OR = 31.07; *P* < 0.001), which probably reflects greater illness severity in those patients. Had we controlled for illness severity, it is likely that the significance of incident delirium as a risk factor for in-hospital mortality would more closely approximate previous reports [7]. 

Like others, we found age and dementia to be the most important risk factors for delirium [27, 28], although longer hospital stays were also associated with incident delirium. In our study, patients with delirium remained in hospital for an average of 9.6 days longer than patients without delirium. Longer hospital stays, however, are also associated with greater illness severity which has also been shown to be an important predictor of delirium [28]. The relative contributions of the two variables to incident delirium cannot, however, be determined in this study as a measurement of the severity of the current illness/condition was not collected. No variables assessed were significantly associated with persistent delirium or readmission to hospital within 28 days following discharge, although the numbers may have been too small to identify important predictors of these outcomes. Of concern, however, almost fifty percent of patients with delirium in this study had persistent delirium, defined as delirium that was still present at the time of discharge. This raises a question about patient safety in the community as persistent delirium is likely to be associated with impaired self-care and poor judgment and places the patient at considerable risk unless they are discharged into a high care environment.

The overall prevalence and incidence of delirium reported in this study are somewhat lower than rates reported previously—both overseas and in Australia [1, 8–11], which vary according to methodological differences, patient characteristics, the diagnostic criteria applied, and whether comorbid conditions including dementia were assessed. For example, the rate of prevalent delirium in this study is lower than the rates reported in two previous Australian studies (18% and 22%) [9, 10], while the rate of incident delirium is both substantially higher and lower than rates previously reported in Australian research (2% and 25%) [9, 11]. While methodological differences are likely to underpin some of the different findings, important differences in patient characteristics across the studies are also likely to account for the different rates. The most important of these is differences in the rates of premorbid cognitive impairment in the patient samples and in comparison to the rate of 20.7% for probably dementia in the present study; rates of 25% were reported in the study by Salih and colleagues [10], and 30.7% in the study by Iseli and colleagues [9], while the patients in the study by Sheng and colleagues [11] were seriously ill older poststroke patients. While our methodology may have resulted in the underestimation of the true prevalence and incidence to a degree (see Section 4.1) the much higher delirium rates in patients with probable dementia in this study which is more consistent with previous findings [1] suggest that any underestimation is likely to be slight. The differing delirium rates reported in patients with and without dementia and cognitive impairment in this and other studies highlight the importance of presenting delirium rates separately for the two groups, which several earlier studies failed to do. 

Finally, we found no difference in the prevalence or incidence of delirium across general medical, general surgical, and orthopedic wards in this study which is inconsistent with previously reported findings of very high delirium rates in older surgical and orthopedic patients [4, 5]. In the present study, however, the results are based on an unselected sample of patients admitted to general surgical and orthopedic wards, of whom a relatively low proportion had surgery (slightly over 20% overall). However, the principal AR-DRG most frequently recorded for patients with delirium admitted to these wards was “other hip and femur procedures” and “hip replacement,” which is consistent with previously reported high rates of both pre- and postoperative delirium in older patients undergoing hip fracture surgery [5]. The very low delirium rate recorded in the hospitals' administrative dataset in this study, as has been reported previously [29], however, suggests a low detection rate by clinicians. This, in turn, suggests that delirium may be undertreated [30].

### 4.1. Study Strengths and Limitations

Strengths of this study include its multicentre, prospective design, relatively large sample size, and chart review by independent physicians. The collection of comprehensive patient data early in the admission means data regarding the patient's cognitive state at admission are likely to be accurate, and the careful monitoring of patients throughout the admission also increases the accuracy of data regarding incident delirium. While chart review by independent physicians allows for a high level of confidence regarding the accuracy of patient diagnoses of dementia and delirium, it nevertheless falls short of “gold standard” face-to-face patient assessment by independent physicians, which was not feasible in this study.

Additional study limitations include the possibility of selection bias from several sources including the requirement that patients have an expected admission of at least 48 hours and the recruitment of patients between Mondays and Fridays only. Another potential source of bias derives from the proportion of patients who declined to participate in the study, although there was no difference in the length of stay between participants and nonparticipants suggesting no difference between the two groups in terms of illness severity. 

Although we aimed to accurately identify dementia and delirium, because not all cases were independently reviewed, the possibility of patient misclassification remains. However, because only a minority of cases were not reviewed (those with the highest MMSE scores), any underestimation is considered to be slight. In addition, some cases were assessed several days following their admission, and in those cases delirium at admission may not have been accurately detected. However, because research physicians had access to all documentation including the patient's medical record, underestimation is again considered to be slight, although it is acknowledged that symptoms of hypoactive delirium are not always recorded in the patient's medical records. 

Furthermore, although the research nurses visited the ward daily during the patient's admission to detect adverse events including delirium, the possibility remains that not all cases of incident delirium were detected. Finally, the exclusion of patients near death is likely to mean that the delirium rate was underestimated as delirium is very common in patients at the end of life [31], and hence our findings are not generalizable to these patients. 

## 5. Conclusion

These data add to the small, but growing, knowledge base in Australia regarding delirium in older patients admitted to general hospitals. It is suggested that hospital clinicians maintain a high index of suspicion for this common and serious condition in older patients, particularly in those with dementia, as some preventive strategies may be effective and may reduce the very substantial financial and clinical burdens associated with the condition.

## Figures and Tables

**Table 1:** Demographic and clinical characteristics and assessment results of patients with delirium versus those without delirium.

Characteristic	Delirium at admission (*n* = 48)^†^; *n* (%)	No delirium at admission (*n* = 445)^†^; *n* (%)	*t*	*χ* ^2^ (df)	*P* value	Incident delirium^††^ (*n* = 34)^†^; *n* (%)	No incident delirium (*n* = 411)^†^; *n* (%)	*t*	*χ* ^2^ (df)	*P* value
Age (years)	M = 84.6; SD = 4.9	M = 79.9; SD = 6.5	−4.8		0.008*	M = 81.2; SD = 6.1	M = 79.8; SD = 6.5	−1.2		1.000
Gender										
Female	26 (54.2)	262 (58.9)				17 (50.0)	245 (59.6)			
Male	22 (45.8)	183 (41.1)		0.39 (1)	1.000	17 (50.0)	166 (40.4)		1.2 (1)	1.000
Diagnosis of dementia	24 (50)	78 (17.5)		27.8 (1)	0.008*	15 (44.1)	63 (15.3)		18.0 (1)	0.023*
Visual impairment	7 (14.6)	39 (8.8)		1.72 (1)	0.865	5 (14.7)	34 (8.3)		1.6 (1)	1.000
Hearing impairment	4 (8.3)	29 (6.5)		0.23 (1)	1.000	5 (14.7)	24 (5.9)		4.0 (1)	0.455
Admitted from										
Private residence	33 (68.8)	398 (89.4)				28 (82.4)	370 (90.0)			
Nursing home										
Low care	4 (8.3)	18 (4.0)				2 (5.9)	16 (3.9)			
High care	10 (20.8)	16 (3.6)				3 (8.8)	13 (3.2)			
Other^‡^	1 (2.1)	13 (2.9)		28.4 (3)	0.008*	1 (2.9)	12 (2.9)		3.1 (3)	1.000
Admitted to										
General medical ward	37 (77.1)	257 (57.8)				18 (52.9)	239 (58.2)			
General surgical ward	7 (14.6)	133 (29.9)				13 (38.2)	120 (29.2)			
Orthopedic ward	4 (8.3)	55 (12.4)		6.9 (2)	0.171	3 (8.8)	52 (12.7)		1.4 (2)	1.000
Did they have surgery requiring general anesthetic^§§^?	7 (14.6)	95 (21.3)		1.2 (1)	1.000	12 (35.3)	83 (20.2)		4.3 (1)	0.455
Most frequent principal diagnosis	Stroke *n* = 12 (25); Hip replacement/other hip and femur procedures *n* = 6 (12.5)	COPD^‡‡^ *n* = 24 (5.4); oesophagitis, gastroenteritis, and other digestive disorders *n* = 19 (4.3)				Hip fracture/other hip and femur procedure *n* = 5 (14.7); heart failure and shock *n* = 4 (11.8)	COPD^‡‡^ *n* = 24 (5.8); oesophagitis, gastroenteritis, and other digestive disorders *n* = 19 (4.6)			

Characteristic	Delirium at admission	No delirium at admission	*t*		*P* value	Incident delirium	No incident delirium	*t*		*P* value

Mini-Mental State Examination; mean (SD)	19.2 (6.6)	25.2 (4.6)	7.2		0.008*	22.3 (5.8)	25.4 (4.4)	3.73		0.023*
Informant Questionnaire for Cognitive Decline in the Elderly (IQCODE); ^§^mean (SD)	4.1 (0.79) (*n* = 25)	3.4 (0.73) (*n* = 144)	−4.1		0.008*	3.7 (0.7) (*n* = 14)	3.4 (0.73) (*n* = 130)	−1.4		0.967
Vulnerable Elders Survey (VES-13); ^¶^mean (SD)	6.64 (3.3)	5.1 (3.2)	−3.04		0.013*	6.3 (2.9)	4.9 (3.3)	−2.5		0.455
Neuropsychiatric Questionnaire (NPI-Q); ^¶¶^mean (SD)	7.1 (5.8) (*n* = 26)	3.4 (4.5) (*n* = 185)	−3.8		0.008*	4.4 (4.4) (*n* = 19)	3.3 (4.5) (*n* = 166)	−1.0		1.000
Charlson Comorbidity Index	4.5 (2.6)	4.0 (2.9)	−1.02		1.000	4.9 (2.4)	3.9 (2.9)	−1.8		0.455

^†^Unless otherwise indicated; ^††^patients with delirium at admission were excluded from the analyses; *P < 0.05.

^‡^Other includes supported accommodation in the community, short-term crisis accommodation, other hospital, and “other”.

^§^IQCODE: higher scores indicate greater decline with scores greater than 3.44 indicative of cognitive decline.

^¶^VES-13 scores of 3 or more in older people indicate an increased risk of functional decline or mortality over the next 2 years.

^¶¶^NPI-Q: scores range from 0 to 36 with higher scores indicative of greater symptomatology.

^§§^GA: general anesthetic; none of the 43 cases assessed following the procedure were considered to have delirium at admission compared to 11.9% (*n* = 7) of those assessed prior to the procedure (*χ* ^2^ = 5.5 (1); *P* = 0.02). ^‡‡^COPD: chronic obstructive airway disease.

**Table 2:** Outcomes of patients with and without delirium at or during the admission.

Outcome	Delirium status	Mean (SD)	*n* (%)	*t*	*χ* ^2^ (df)	*P* value
Length of stay in hospital; mean (SD)	Delirium at admission (*n* = 48)	15.9 days (14.3)		−3.9		0.005*
	No delirium (*n* = 445)	9.7 days (9.7)				
	Incident delirium (*n* = 34)^†^	21.7 days (17.9)		−8.0		0.005*
	No delirium (*n* = 411)	8.66 days (8.0)				

Number of those who fell in hospital	Delirium at admission		6 (17.1)		12.3 (1)	0.005*
	No delirium		18 (3.9)			
	Incident delirium		5 (14.7)		11.9 (1)	0.005*
	No delirium		12 (2.9)			

In-hospital mortality	Delirium at admission		4 (8.3)		5.8 (1)	0.073
	No delirium		10 (2.2)			
	Incident delirium		8 (23.5)		75.9 (1)	0.005*
	No delirium		2 (0.5)			

Deceased at 28 days after discharge^#^	Delirium at admission		5 (13.5)		19.8 (1)	0.005*
	No delirium		6 (1.5)			
	Incident delirium		2 (7.7)		7.2 (1)	0.029*
	No delirium		4 (1.1)			

Discharged to a higher care level^§^	Delirium at admission		6 (20.0)		10.4 (1)	0.005*
	No delirium		19 (5.1)			
	Incident delirium		3 (15.8)		4.6 (1)	0.08
	No delirium		16 (4.6)			

Readmitted to hospital within 28 days after discharge	Delirium at admission		2 (5.0)		3.08 (1)	0.260
	No delirium		62 (15.2)			
	Incident delirium		5 (14.7)		0.006 (1)	1.000
	No delirium		57 (15.2)			

^†^Delirium was not present at admission; ^#^there was no significant difference in mortality at 28 days according to whether delirium had resolved at discharge or not (*χ* ^2^ (1) = 0.814; *P* = 0.37).

^§^Higher level of care is defined as patients newly admitted to a nursing home when they had been previously living in the community prior to the hospital admission and patients who transferred from low to high nursing home care following hospitalization. *P < 0.05.

**Table 3:** Significant predictors of delirium and adverse outcomes in hospitalized older patients.

Outcome	Predictor	Odds ratio	95% confidence interval	*P* value
Delirium at admission	Diagnosis of dementia	3.18	1.65–6.14	<0.001**
	Age	1.09	1.03–1.14	<0.001**

Incident delirium	Diagnosis of dementia	4.82	2.19–10.62	<0.001**
	Length of stay	1.08	1.05–1.12	<0.001**

Delirium not resolved by discharge	No variables significant			

Falls in hospital	Length of stay	1.07	1.05–1.11	<0.001**
	Delirium at admission	2.83	0.99–8.02	0.050

In-hospital mortality	Delirium at admission	5.19	1.27–21.24	0.022*
	Incident delirium	31.07^†^	9.30–103.78	<0.001**

Deceased at 28 days of followup	VES-13 score	1.71	1.14–2.57	0.009*
	Length of stay	1.04	1.00–1.08	0.034*

Discharged to a higher care level	Length of stay	1.10	1.07–1.14	<0.001**
	Age	1.14	1.06–1.23	0.001*
	Male gender	4.18	1.16–15.09	0.029*

Readmission to hospital within 28 days after discharge	No variables significant			

^†^Of the 14 patients who died in hospital, 9 had incident delirium. *P < 0.05; **P < 0.001.
